# Beyond Nyquist: A Comparative Analysis of 3D Deep Learning Models Enhancing MRI Resolution

**DOI:** 10.3390/jimaging10090207

**Published:** 2024-08-23

**Authors:** Soumick Chatterjee, Alessandro Sciarra, Max Dünnwald, Anitha Bhat Talagini Ashoka, Mayura Gurjar Cheepinahalli Vasudeva, Shudarsan Saravanan, Venkatesh Thirugnana Sambandham, Pavan Tummala, Steffen Oeltze-Jafra, Oliver Speck, Andreas Nürnberger

**Affiliations:** 1 Data and Knowledge Engineering Group, Otto von Guericke University Magdeburg, 39106 Magdeburg, Germany; 2 Faculty of Computer Science, Otto von Guericke University Magdeburg, 39106 Magdeburg, Germany; max.duennwald@med.ovgu.de (M.D.); anitha.bhat.talagini.ashoka@idmt.fraunhofer.de (A.B.T.A.); magumayur.gurjar@gmail.com (M.G.C.V.); shudarsansaravanan@gmail.com (S.S.); venkatesh.thirugnana@ovgu.de (V.T.S.); pavan-s9@outlook.com (P.T.); 3 Genomics Research Centre, Human Technopole, 20157 Milan, Italy; 4 Department of Biomedical Magnetic Resonance, Otto von Guericke University Magdeburg, 39106 Magdeburg, Germany; alessandro.sciarra@ovgu.de (A.S.); oliver.speck@ovgu.de (O.S.); 5 MedDigit, Department of Neurology, Medical Faculty, University Hospital Magdeburg, 39120 Magdeburg, Germany; oeltze-jafra.steffen@mh-hannover.de; 6 Fraunhofer Institute for Digital Media Technology, 98693 Ilmenau, Germany; 7 German Centre for Neurodegenerative Diseases, 37075 Magdeburg, Germany; 8 Centre for Behavioural Brain Sciences, 39106 Magdeburg, Germany; 9 Peter L. Reichertz Institute for Medical Informatics, Hannover Medical School, 30625 Hannover, Germany

**Keywords:** MRI, undersampling, super-resolution, deep learning

## Abstract

High-spatial resolution MRI produces abundant structural information, enabling highly accurate clinical diagnosis and image-guided therapeutics. However, the acquisition of high-spatial resolution MRI data typically can come at the expense of less spatial coverage, lower signal-to-noise ratio (SNR), and longer scan time due to physical, physiological and hardware limitations. In order to overcome these limitations, super-resolution MRI deep-learning-based techniques can be utilised. In this work, different state-of-the-art 3D convolution neural network models for super resolution (RRDB, SPSR, UNet, UNet-MSS and ShuffleUNet) were compared for the super-resolution task with the goal of finding the best model in terms of performance and robustness. The public IXI dataset (only structural images) was used. Data were artificially downsampled to obtain lower-resolution spatial MRIs (downsampling factor varying from 8 to 64). When assessing performance using the SSIM metric in the test set, all models performed well. In particular, regardless of the downsampling factor, the UNet consistently obtained the top results. On the other hand, the SPSR model consistently performed worse. In conclusion, UNet and UNet-MSS achieved overall top performances while RRDB performed relatively poorly compared to the other models.

## 1. Introduction

Magnetic resonance imaging (MRI) with high-spatial resolution provides rich structural information, which facilitates effective clinical diagnosis, decision-making, and precise quantitative image analysis. Nevertheless, with MRI being an inherently slow process, high-resolution scans result in prolonged scan time due to hardware and physical constraints. Undersampling can accelerate scan times, but it comes with the trade-off of slightly lower-resolution images and the potential presence of artefacts. Reconstructing a high-resolution (HR) image from a low-resolution (LR) input, it is possible to achieve larger spatial coverage, higher SNR and resolution in a shorter scan time [1]. The classical basic approach consists of the interpolation of LR images into HR. However, the interpolation methods fail to recover the loss of high-frequency information such as fine edges of objects, and also make it very challenging to restore texture and structural details accurately. Another approach is to scan multiple LR images and fuse them into a single HR image. Nevertheless, this is not very robust to inter-scan motion and is neither time- nor cost-efficient in practice. Therefore, single image super-resolution (SISR) [2] is a desirable approach, as it requires only one LR scan to produce an HR output without additional scan time.

SISR is an optimisation problem and consists of minimising the cost function between the observed LR image and the model estimation, with the aid of regularisation terms. However, regularisation terms require a priori image distribution knowledge, which is often based on empirical assumptions. Common constraints such as total variation implicitly assume that the image is piece-wise constant, which is problematic for images with fine structures and many local details. On the other hand, learning-based approaches do not need such well-defined priors. In particular, deep-learning-based techniques have shown great improvement in the SISR task, even for images with rich details, due to their nonlinearity and remarkable ability to emulate accurate transformation between LR and HR in challenging cases. Super-Resolution Convolutional Neural Networks (SRCNN) [3] and Faster-SRCNN (FSRCNN) [4] demonstrated tremendous potential and outstanding results for 2D natural images.

In MRI, the type and amount of image degradation depend on the type of undersampling and the undersampling factor. If the image is undersampled using sampling techniques, such as variable density sampling, uniform sampling, radial sampling, the task of reconstruction of the undersampled images can be treated as an artefact reduction task. On the other hand, MRIs with low-spatial resolution can be improved by treating it as a super-resolution task. Deep-learning-based techniques to improve the image quality of MRIs have been proposed for both artefact reduction [5,6] and super-resolution [7,8,9]. The focus of this research is on the latter, improving the image quality of low-resolution MRI by treating it as an SISR problem.

It is important to note that many medical images are 3D volumes, and the 2D super-resolution networks work slice-by-slice without exploiting the full advantage of continuous structure in 3D. Two-dimensional networks stack multiple slices on top of each other, eliminating the continuous structure information from the third dimension. The direct conversion of a 2D approach into 3D can result in a large number of parameters and therefore pose challenges in memory allocation, while these 2D-adapted deep learning approaches do not fully address the medical image SR problem [10]. Therefore, a 3D model would be more preferable to directly extract 3D features, considering the object across multiple slices.

### 1.1. Contributions

In this work, the performance of different 3D super-resolution deep learning models applied to MR brain images have been evaluated. This includes two models proposed for the task of super-resolution: Structure Preserving Super Resolution model (SPSR) [11] and ShuffleUNet [8], two models that were proposed for image segmentation and suitable for inverse problems: UNet [12] and UNetMSS [13,14], and finally, a custom in-house model taking the building blocks of SPSR: Residual in Residual Dense Block model (RRDB). The aim is to evaluate the different models for various down-sampling factors ranging from 8 to 64, and MR contrasts $T_{1}$, $T_{2}$, PD weighted images. Additionally, cross-contrast cross-resolution experiments were performed that have not been found in the literature so far. Moreover, the models were compared in terms of the number of trainable parameters (i.e., model complexity) and the time required for inference. The inference time is essential as this provides additional overhead for the final reconstruction. Finally, an uncertainty evaluation framework was developed and the models were compared in terms of uncertainty—as in the field of medical imaging, building trust of the medical professionals in the models we are using is important.

### 1.2. Background

As stated above, the aim of this work was to compare the performances of different deep learning models when transforming LR into HR images. The mathematical relation between them can be written as:

(1) y=f(X)

where *f* is an arbitrary continuous transformation function that downgrades the image *X*.

The aim of the super-resolution task is to find the inverse function g(Y), which is almost equal to $f^{-1}\left(Y\right)$. It can be shown that:

(2) $$\hat{X}=g\left(Y\right)=f^{-1}\left(Y\right)+r$$

where *r* is the reconstruction residual. A learning-based SR process is based on three steps in order to restore *X*:

1. Extract image features from *Y*
2. Map the feature vector to a feature space
3. Reconstruct *X* from the feature space

Convolutional neural networks have the ability to handle these steps clearly [3] by minimising the difference between reconstructed images and ground-truth images during the training process.

## 2. Methods

### 2.1. Dataset

This research utilised the publicly available IXI dataset (IXI Dataset: https://brain-development.org/ixi-dataset/ accessed on 17 August 2024), which is a collection of TI, T2, and PD-weighted images, MRA images, and diffusion-weighted images of nearly 600 healthy subjects, collected from Hammersmith Hospital using a Philips 3T system, Guy’s Hospital using a Philips 1.5T system, and the Institute of Psychiatry using a GE 1.5T system. The first set of experiments in this research was performed using T1-weighted images, while subsequent experiments also encompassed T2 and PD -weighted images. The MR volumes possessed an average voxel size of approximately 0.9×0.9×1.2.

#### Undersampling

The MRIs from the dataset were considered as fully sampled, high-resolution ground-truth images, which were subsequently undersampled in all three spatial dimensions to generate the low-resolution images serving as the inputs for the models. The downsampling of these high-resolution images, in all three directions, was performed using the resample function of the FSLPy library [15], which uses sinc interpolation. This procedure was carried out with scale factors of $2^{3}$, $2.5^{3}$, $3^{3}$, $3.5^{3}$, and $4^{3}$, resulting in theoretical acceleration factors of 8, ∼16, 27, ∼43, 64, respectively.

### 2.2. Network Models

This research utilised five different network models, among which two were specifically proposed for super-resolution: Structure Preserving Super Resolution (SPSR) [11] and ShuffleUNet [8], two generic deep learning models that are widely employed to address inverse problems: UNet [12] and UNetMSS [13,14], and finally, the building block of SPSR: Residual in Residual Dense Block (RRDB).

#### 2.2.1. Residual in Residual Dense Block (RRDB)

**Figure 1:**
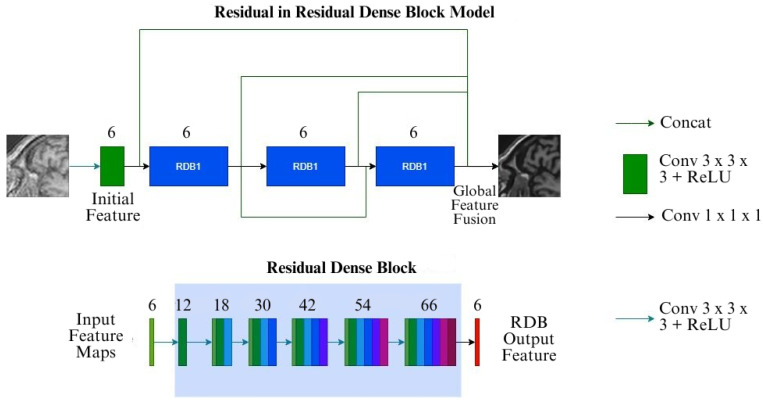
Schematics of the Proposed RRDB Architecture, the input image passes through a shallow feature extractor followed by a series of Residual Dense Blocks (RDB). In each layer of the RDB, the features of all previous layers are cascaded and in the end the cascaded features are compressed using 1 × 1 Convolution operations. After a series of RDB, the outputs from all the RDBs are fused together to generate the SR images.

DenseNet [16] is a well-established network model, primarily used for image classification tasks. Taking inspiration from the DenseNet architecture, Wang et al. introduced Residual in Residual Dense Block (RRDB) for their GAN-based super-resolution framework ESRGAN [17]. Due to their additional layer of training complexity, GANs were not considered in this current research, but the RRDB was included in the set of models. The version of RRDB employed here in this research includes three dense blocks with six layers in each of them with residual connections, a growth rate of 12 with four initial feature maps. The output feature maps from the dense block are then consolidated together with a 1 × 1 × 1 convolution, and this gives the output of the model. The model architecture is shown in Figure 1.

#### 2.2.2. Structure Preserving Super Resolution (SPSR)

**Figure 2:**
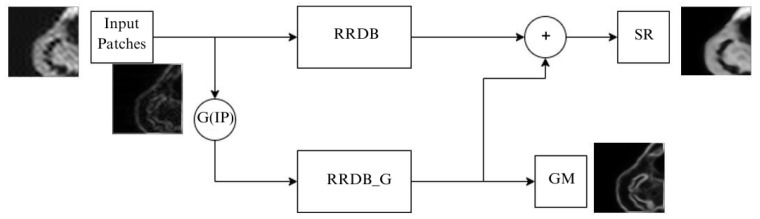
Structure preserving super-resolution framework (SPSR), contains two parallel RRDB, the base RRDB extracts features from the images and the gradient RRDB (RRDBG) extracts features from the gradients of the images. The features from both the branches are fused together to obtain SR images.

Many super-resolution approaches result in blurry reconstructions and produce a statistical average of the dataset. Some of the GAN-based methods can produce reasonable reconstructions, but GAN-based methods are difficult to train and interpret. Structure preserving Super Resolution (SPSR) is a technique that attempts to preserve structures in the reconstruction without employing a GAN-based framework [11]. It uses a dual-branch approach, where the super-resolution branch attempts to super-resolve the image by stacking RRDBs, and uses a gradient branch attempts to super-resolve the gradient of the low-resolution image into the gradient of the high-resolution image. The gradient branch attempts to predict a gradient map for the high-resolution image, which is used to recover the sharpness and structure of the image. The gradient maps of the low-resolution input and the high-resolution ground-truth are generated by passing a Sobel filter over the images. The recovered gradients can be integrated in the results of the first branch to provide a structural prior for the super-resolved image. There is a gradient loss that monitors the prediction of the gradient maps. Along with the image space loss functions, the gradient loss restricts the relationships with the neighbouring pixels. The authors demonstrate that this preserves structural consistency after super-resolving. Figure 2 shows the SPSR framework.

#### 2.2.3. U-Net

**Figure 3:**
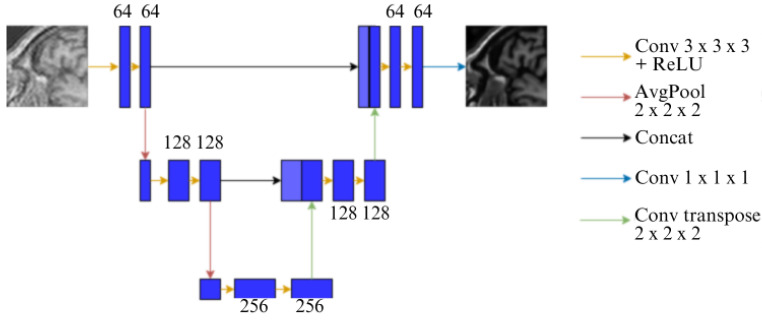
Conventional U-Net architecture.

The UNet model (shown in Figure 3), one of the most popular models for inverse problems, was originally proposed for the task of image segmentation [12]. There are two paths: a downsampling and an upsampling. In the downsampling path, also called the contracting path, is a classic convolutional network, where repeated convolutions, followed by ReLU and MaxPool operations take place. In each step, the number of feature channels is generally doubled. During this downsampling process, the spatial information is reduced and the feature information is increased. In the upsampling path, also called the expansion path, the upsampling of the feature map takes place, while halving the number of feature channels by the process of up-convolution, along with concatenation with those comparable feature maps from the downsampling path.

#### 2.2.4. U-Net MSS

**Figure 4:**
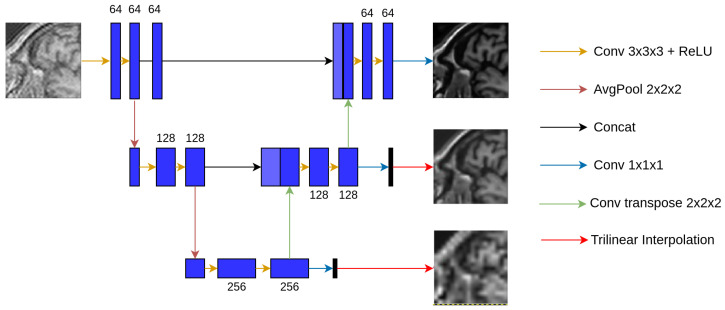
UNet-MSS.

U-Net with Multi-Scale Supervision (MSS) [13] is a version of the U-Net model that uses an architecture identical to U-Net, except for the loss term. The loss for U-Net is calculated by comparing its output obtained from the final scale of the model against the ground-truth, while the loss for U-Net MSS is computed at different scales. This is performed by taking output of the different blocks of the expansion path, resulting in outputs of different scales, then were interpolated using nearest-neighbour interpolation to have the same size as the ground-truth, before finally calculating the loss at different scales by comparing these interpolated outputs against the ground-truth. Individual loss values obtained at different scales are then summed up and backpropagated to train the model. This reassures the learning of discriminative features at each level of upsampling and should also enhance the learning by allowing easier gradient flow to the earlier blocks of the network [14]. The total loss of this model is calculated following Equation (3) and the loss calculation mechanism is depicted in Figure 4.

(3) $$L_{MSS}\left(\theta \right)=\frac{1}{\sum_{i=1}^{s}\alpha_{i}}\sum_{i=1}^{s}\alpha_{i}l_{scale}^{i}\left(\theta \right)$$

where *s* denotes the number of scales where the loss is computed, $l_{scale}^{i}$ represents the loss (using any given loss function) at scale *i*, $\alpha_{i}$ represents the weight for that particular scale, and finally, θ denotes the network parameters.

#### 2.2.5. ShuffleUNet

The ShuffleUNet ([8]), just like any UNet-like architecture, has two paths: the contraction path and the expansion path. Contraction Path consists of 4 blocks, each of them down samples the input by half in all dimensions. Each block in the contraction path consists of three sub-blocks:

1. Double convolution
2. Convolutional decomposition
3. Pixel unshuffle

The input goes to double convolution, and its output serves as the input of the convolutional decomposition sub-block. The input of this sub-block is provided to every convolution of this block and four different outputs are obtained—these outputs are cited as convolutional decomposition of the input of this sub-block. Pseudo-lossless downsampling operation, pixel unshuffle is applied on the fourth output, which down-samples the input by an element of two all told dimensions, therefore the remainder of the outputs are directly forwarded as skip-connection to the expansion path.

At the end of the contraction path, one final double convolution is applied to the output of last unshuffle as the latent convolution. This output is then sent to the expansion path.

The expansion path also consists of four blocks each of which up samples by two factors in all dimensions, and these blocks have three sub-blocks namely:

1. Pixel Shuffle
2. Convolutional decomposition
3. Double Convolution

**Figure 5:**
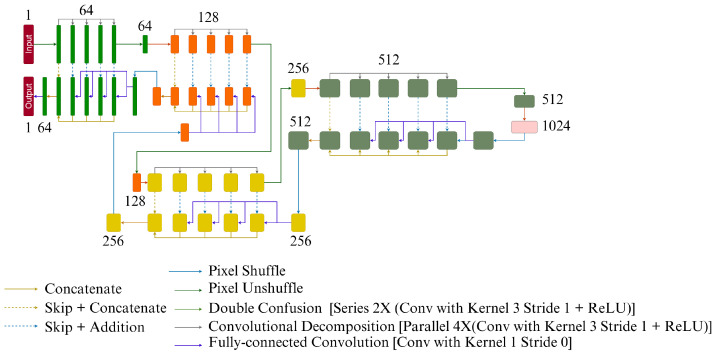
ShuffleUNet.

The pixel shuffle up scales the input, and it is up sampled using periodic shuffling operation in feature space. The output of this block is passed on to the convolutional decomposition to generate four different outputs which are added to the incoming skip connections from the same level as of the contraction path. Concatenation takes place together with the output of the skip connection coming from the pixel unshuffle operation, which is then forwarded to the double convolution sub-block. The final output is the fully connected convolution layer. Figure 5 shows the typical structure of a ShuffleUNet.

### 2.3. Implementation, Training, and Evaluation

#### 2.3.1. Dataset Split

The dataset was split (see Table 1) into training, testing, and validation sets in the following manner:

1. Training—70 percent of the entire data set
2. Testing and Validation—30 percent of the entire data set
  (a) Testing—60 percent of the remaining 30 percent data set.
  (b) Validation—40 percent of the remaining 30 percent data set.

In total, 1212 images were used for training, 210 for validation, and 313 for the test.

To train the scale generic models across all give acceleration factors, same split proportions of the images were used, but for the scale factor, a random scale factor was picked during every epoch for every image. The probability distribution for this selection is uniform, so that there is no bias in the model for one specific acceleration factor.

#### 2.3.2. 3D Image Patching and Merging

The authors used multiple patch samples from a volume for training to make the process computationally favourable. Multiprocessing CPU techniques were adopted in parallel for training iterations. As and when the volumes are pre-processed and these training ready samples are sorted in the queue until the next training iteration. They are loaded into the GPU for inference. This research utilised TorchIO [18] which provides the queue class, which is inherited from the PyTorch Dataset [19]. In this queuing system, samplers behave as generators that yield patches from random locations in volumes contained in the SubjectsDataset.

Sinc interpolation, a commonly used interpolation technique in MRI, was used for preprocessing the images; this method of interpolating the image data by zero filling the high spatial frequency components of the raw data so that after Fourier transformation the image matrix size has been increased. This method helps to improve the image display quality. For the experiments, a lazy interpolation on LR images was performed, that is, the interpolated images are generated before hand and stored as NIFTI files. TorchIO reads this file creates a data loader object and then used this data loader object to train the models (https://torchio.readthedocs.io/patches/patch_training.html accessed on 17 August 2024).

#### 2.3.3. Training

At the beginning of each training epoch, the subject list in the subjects dataset is shuffled, to increase the variance of training instances during model optimisation. The PyTorch loader queries the copied datasets in each process, which load and process volumes in parallel on the CPU. A patch list is prepared, consisting of patches from different subjects. To make each batch consisting of different subjects, the queue gets shuffled once it reaches the maximum length. Using multiprocessing, the internal data loader queues the subjects dataset continuously. When emptied, the patch list is refilled with new patches. A second data loader, external to the queue, may be used to collate batches of patches stored in the queue, which are passed to the neural network. Each 3D image is split into 64 cubes. The location of the cube is selected randomly for training. Torchio’s UniformSampler was used here, which randomly extracts patches from a volume with uniform probability.

Torchio’s Grid sampler was employed to perform inference using all patches from a volume, and the grid aggregator was used for merging the 3D cubes back to the whole image. The “Average” overlap mode of the grid aggregator was utilised, so that the predictions in the overlapping areas will be averaged with equal weights.

As input to the model for training purposes, different acceleration factor images were provided. The acceleration factors were five discrete scaling factors on all sides: 2 fold, 2.5 fold, 3 fold, 3.5 fold, 4 fold, respectively. The weights for all the scaling factors are the same. The images are broken into patches and fed into the model for training and validation purposes. When patches from all the subjects are used for training then its defined as the end of a training.

A 64 × 64 × 64 sized image has 48 unique patches without overlap. For all experiments, 60 patches were taken from each volume, and all voxel intensities were re-scaled between 0 and 1. There were no other pre-processing or data augmentation involved. The learning rate is reduced on plateau with patience of three epochs. All experiments were performed by optimising the loss terms with a learning rate of 0.0001 using the Adam optimiser [20] with an effective batch size of 22 for 50 epochs.

The aim of weight initialisation is to prevent the output layers from exploding or disappearing during the feed-forward of a neural network. If any of the other occurs, the network might take much longer to converge. There are multiple approaches to initialise weights that prevent them from exploding or vanishing. In this research, the Xavier Glorot uniform distribution was used that sets a layer weights to values chosen from a random uniform distribution that is bounded between weights.

(4) $$W_{ij}∼U\left[-\frac{1}{\sqrt{n}},\frac{1}{\sqrt{n}}\right]$$

where *U* is a uniform distribution and *n* is the size of the previous layer (number of columns in *W*).

It is believed that this weight initialisation would maintain the variance of activations and backpropagated gradients all the way.

All experiments are made reproducible by having a constant random seed value. The validation step is performed after every epoch, and the best model weight is saved based on the validation loss. Validation is basically used to monitor the training process. After every training epoch, validation loss is obtained from the validation set to check whether the model has converged or not. During this phase is when the model is checked for overfitting.

#### 2.3.4. Evaluation

During training, the weights for the best loss in the validation set were used to evaluate the corresponding models. Evaluation metrics were calculated for full images and not on patches. In order to avoid edge artefacts due to patching and restitching, the images are created with an overlap with average mode while restitching the patches.

This research used mean squared error (MSE), root mean square error (RMSE), peak signal-to-noise ratio (PSNR), and structural similarity (SSIM) [21] metrics to monitor the training process and to evaluate the trained models.

#### 2.3.5. Loss Functions

Loss functions are one of the important components of any machine learning algorithm. Loss functions are used to find the loss (or error) between the current output and the expected output. These provide a quantifiable measure of how far off the output from the expectations is. Choosing a proper loss function that will be able to give almost exact error is an important task in any deep learning project. In order to find the best, experiments were performed with five different loss functions; SSIM (Structural Similarity Index) [21], Perceptual SSIM loss, mean absolute error (L1), perceptual L1 loss, and mixed gradient loss (MGL). Among them, the authors found that SSIM loss performs the best.

##### Structural Similarity Index

Structural similarity index or SSIM [17,22], is a metric to measure the similarity of the two images. SSIM is calculated over the corresponding windows of an image, at its low and high resolutions. SSIM measures between two windows of size N × N of an image consider a as output and b as expected (ground truth) is given in the equation:

(5) $$SSIM\left(a,b\right)=\frac{\left(2\mu_{a}\mu_{b}+c_{1}\right)\left(2\sigma_{ab}+c_{2}\right)}{\left(\mu_{a}^{2}+\mu_{b}^{2}+c_{1}\right)\left(\sigma_{a}^{2}+\sigma_{b}^{2}+c_{2}\right)}$$

where $\mu_{a}$ is the average of *a*, $\mu_{b}$ is the average of *b*, $\sigma_{a}^{2}$ is the variance of *a*, $\sigma_{b}^{2}$ is the variance of *b*, $\sigma_{ab}$ is the covariance of *a* and *b*, the constants $c_{1}={\left(k_{1}L\right)}^{2}$, $c_{2}={\left(k_{2}L\right)}^{2}$ are used to stabilise when the denominator tends to zero and *L* is the dynamic range of the values of the pixels.

##### Mean Absolute Error (L1)

Also called the least absolute deviations or L1 ([23]) loss function, it reveals the absolute differences between the output image and the expected image. It sums all the absolute differences between the two images by measuring the average weight of the errors in a set of predictions. It is a robust loss function, but might end up giving one or more solutions. The L1 measure between two images x and y is given in the equation:

(6) $$L1\left(a,b\right)=\sum_{i=1}^{n}\left|a_{i}-b_{i}\right|$$

where $a_{i}$ is the output value and $b_{i}$ is the expected value.

##### Perceptual Loss

While comparing two images that are very similar but differ by shifted pixels, perceptual loss functions can be used. These loss functions use differences, such as style discrepancies or content between comparable images. These losses take the mean of the sum of all squared errors between all pixels, unlike per-pixel loss functions, which use the sum of absolute errors between pixels [24].

In this research, Perceptual SSIM and Perceptual L1 losses were evaluated. The perceptual loss L1 uses the loss L1 to compare the features that were generated by the model on ground truth images and super-resolved images, and perceptual-SSIM loss represents the SSIM loss which is used to compare the features that were generated by the model on ground truth images and super-resolved images. To calculate perceptual losses, the features of the contraction path of the model which was proposed in the DS6 paper [14], were used.

During the initial experiments, it was found that using these perceptual losses was computationally expensive, and each epoch would take more than 26 h at the least, and hence they were avoided in the further experiments.

##### Mixed Gradient Loss ([25])

Mean Square Error, also called as L2 loss function, is another commonly used loss function. It measures the average of squares of pixel errors between two images a and b. It is given by (for a two-dimensional image):

(7) $$MSE\left(a,b\right)=\frac{1}{l\;\cdot \;m}\sum_{i=1}^{l}\sum_{j=1}^{m}{\left(a_{i}{,}_{j}-b_{i}{,}_{j}\right)}^{2}$$

where $a_{i}{,}_{j}$ is the output value, $b_{i}{,}_{j}$ is the expected value, *l* is the number of horizontal pixels, and *m* is the number of vertical pixels.

However, using this loss function alone would not eliminate the gradient error measurement problem. To solve this, the classic gradients are added to the loss function, by using the Sobel operator. That is, for the expected image b:

(8) $$\begin{array}{c} G_{x}=b*\left[\begin{array}{ccc} -1 & -2 & -1\\ 0 & 0 & 0\\ 1 & 2 & 1 \end{array}\right] \end{array}$$

(9) $$\begin{array}{c} G_{y}=b*\left[\begin{array}{ccc} -1 & 0 & 1\\ -2 & 0 & 2\\ -1 & 0 & 1 \end{array}\right] \end{array}$$

where $G_{x}$ is the gradient map *G* of the expected image *b* in the *x*-direction and $G_{y}$ is the gradient map *G* of the expected image *b* in the *y*-direction.

Then, the gradient values are combined in all directions using:

(10) $$G\left(i,j\right)=\sqrt{G_{x}^{2}\left(i,j\right)+G_{y}^{2}\left(i,j\right)}$$

Similarly, the gradient map $\hat{G}$ for the output image a can be calculated.

Next, the mean gradient error (mGE) was computed as:

(11) $$mGE=\frac{1}{l}\frac{1}{m}\sum_{i=1}^{l}\sum_{j=1}^{m}{\left(G_{i}{,}_{j}-{\hat{G}}_{i}{,}_{j}\right)}^{2}$$

Later, the mixed gradient loss (MGL) was computed by adding mean gradient error to mean square error by means of a weight $\lambda_{G}$.

(12) $$MGL=MSE+\lambda_{G}mGE$$

Similarly, the mixed gradient loss can be calculated for a three-dimensional image by adding the parameters in the *z*-direction.

### 2.4. Uncertainty Mapping

After training and evaluating all models, an auxiliary system was built to help the user determine the robustness of the model in generating the super-resolved images. Medical imaging is a risk-sensitive field; therefore, the reliability and robustness of a model are more important than just looking at the final resultant metrics. There have been several approaches to estimate this uncertainty in model predictions. Some methods involve producing natural distributions on the possible predictions by probabilistic formulation over the model parameters, and some exploit the randomness that occurs due to training or inference-time perturbations such as dropouts to estimate the uncertainty in the model predictions [26]. The problem with all these approaches is that it requires these models to be re-trained again, which increases the computational overload significantly. Hence, attention has been given to methods that do not require any training to estimate this uncertainty. Ref. [27] proposed a method that does not involve training exclusively for uncertainty estimation and mapping. There are basically 2 methods which were discussed.

- Black-box case: Where it is not possible to access to all the model parameters and its internal structure. For these cases the authors proposed an infer-transformation based uncertainty estimation where the images were given tolerable transformations across different dimensions, i.e., random flips and rotations which does not involve major structural changes in the images. Since this research uses anisotropic images where the voxel sizes varies across different dimensions the images there are heavy perturbations across other dimensions if one dimension is flipped. This altered the structure of the MRIs.
- Grey-box case: This is a case where access to the model structure, the authors propose to introduce an internal embedding/representation manipulation say by introducing a dropout and a noise layers during the inference time. This is feasible, since there is the independence to alter the model structure as well as the model weights.

The authors tested the final generic RRDB Section 2.2.1 and UNetMSS Section 2.2.4 models with the greybox methods, and trained them on all three IXI contrasts (T1, T2, and PD) for images that are downsampled in all three directions, resulting in the theoretical acceleration factors of 8, ∼16, 27, ∼43, 64, respectively. The objective here is to add tolerable perturbation to the features generated in the intermediate layers by adding either dropouts or noise layers and generate multiple images with different parameters in either of them i.e, dropout rate in the case of infer-dropout and Gaussian noise sigma in case of infer-noise. The final uncertainty map is the voxel-wise variance across all these images.

#### Uncertainty Mapping Pipeline

**Figure 6:**
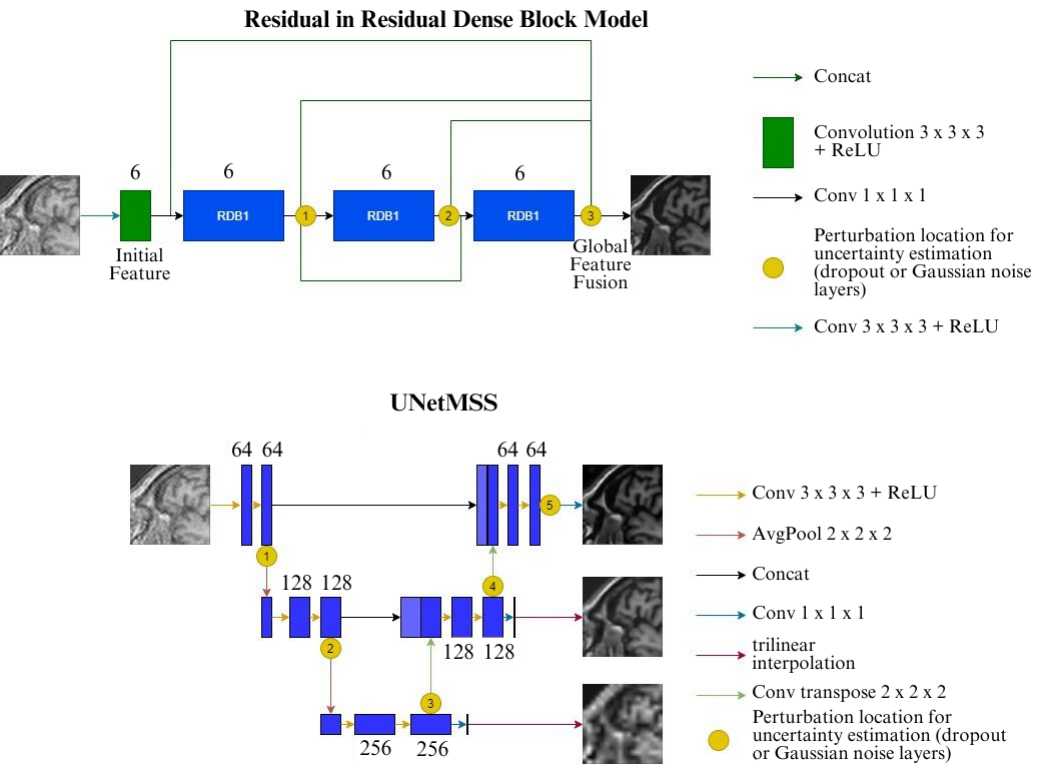
RRDB and UNet (as well as UNetMSS) models with locations where the feature map perturbations are given.

As mentioned in Section 2.4, the grey box methods that were experimented with in this research are infer-noise and infer-dropout methods on the generic RRDB models. The locations in which the perturbations of the feature map are given can be found in Figure 6. In case of RRDB there were three locations at the end of each Residual Dense Block. In case of UNetMSS there were five locations, after each down-path and up-path block. The hyper parameters, i.e., dropouts for infer-dropout and sigma for infer-noise were the same across all these locations. The different dropout rates used for the infer-dropout methods are {0.01, 0.02, 0.05, 0.1, 0.2, 0.5} and the same parameters are used as Gaussian sigma parameter in the infer noise method. The steps involved in the generation of the uncertainty maps are as follows.

1. Iteratively, model predictions are generated from low-resolution images using the trained model weight but with different dropout rates and Gaussian noise in the intermediate layers.
2. Compile all the generated images.
3. The uncertainty map is the pixel-wise variance across all these generated images.

The initial results of this uncertainty mapping pipeline have previously been presented at ISMRM 2022 [28].

## 3. Experiments and Evaluation

### 3.1. Experiments

This section elaborately explains the results obtained from the different experiments conducted during the course of this research. The experiments are categorised into two categories:

1. Initial Experiments
2. Main experiments

Initial experiments are performed to choose the optimum loss function and to finalise the three best performing models for further generalisation experiments.

The main experiments are to find the generalisability of the chosen models and to find the uncertainty mapping for them.

#### 3.1.1. Initial Experiments

The list of experiments considered within the initial experiments is as follows:

1. Different Loss function result comparison.
2. Different 3D CNN models results comparison for cross acceleration factor on IXI-T1 dataset.
3. Models comparison on trainable parameters and inference time.
4. Visualising individual model results for all the acceleration factors.

##### Different 3D CNN Models Results Comparison for Cross Acceleration Factor of IXI-T1 Dataset

In real-time clinical applications, different acceleration factors are used depending upon the task at hand, as well as due to subject-related requirements. Hence, the authors conducted experiments to understand the generalisability of the models in terms of acceleration factors with a scale generic approach, where the authors trained models that could handle all these discrete acceleration factors mentioned before. Hence, all models were trained for five different acceleration factors (i.e, $2^{3}$, $2.5^{3}$, $3^{3}$, $3.5^{3}$, $4^{3}$) in the IXI-T1 dataset. All models were trained with same hyperparameters except for the batch size, since some of the models like SPSR, ShuflleUNet are memory heavy. The training and evaluation process is explained in Section 2.3. This limited batch size problem was tackled with gradient accumulation, where multiple forward passes are performed, the gradients are accumulated, and then backpropagated with the accumulated gradients.

All five deep learning models performed better compared to traditional interpolation-based methods like bicubic interpolation, nearest neighbour interpolation and sinc interpolation.

**Figure 7:**
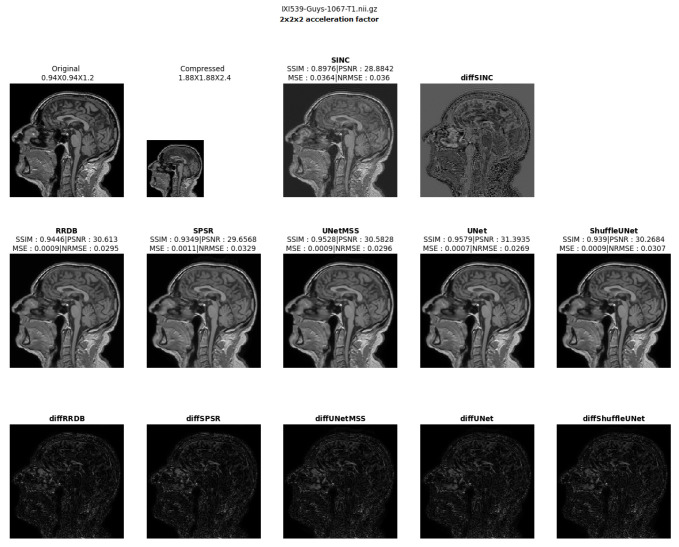
Model comparison for acceleration factor of $2^{3}$ (IXI-T1 dataset).

**Figure 8:**
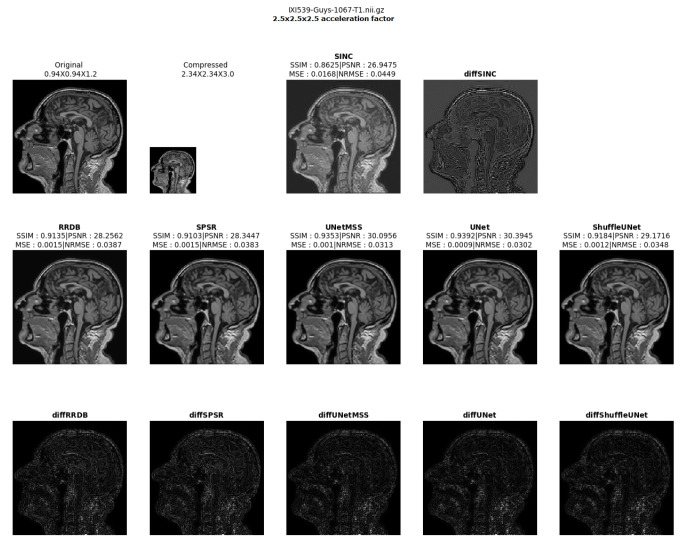
Model comparison for acceleration factor of $2.5^{3}$ (IXI-T1 dataset).

**Figure 9:**
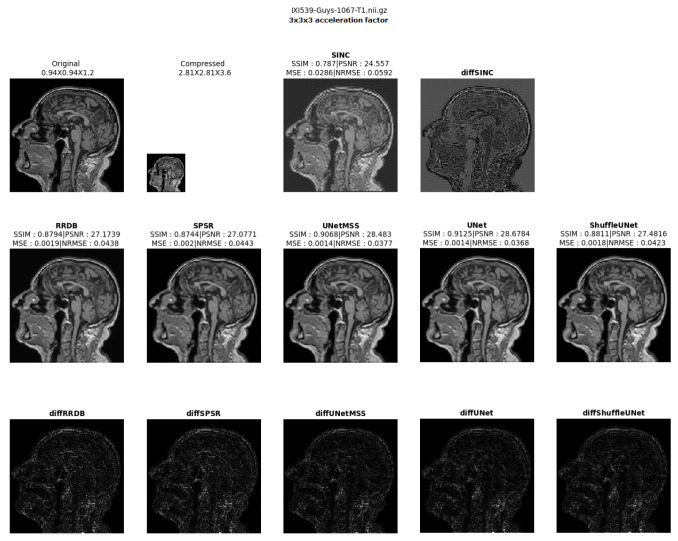
Model comparison for acceleration factor of $3^{3}$ (IXI-T1 dataset).

**Figure 10:**
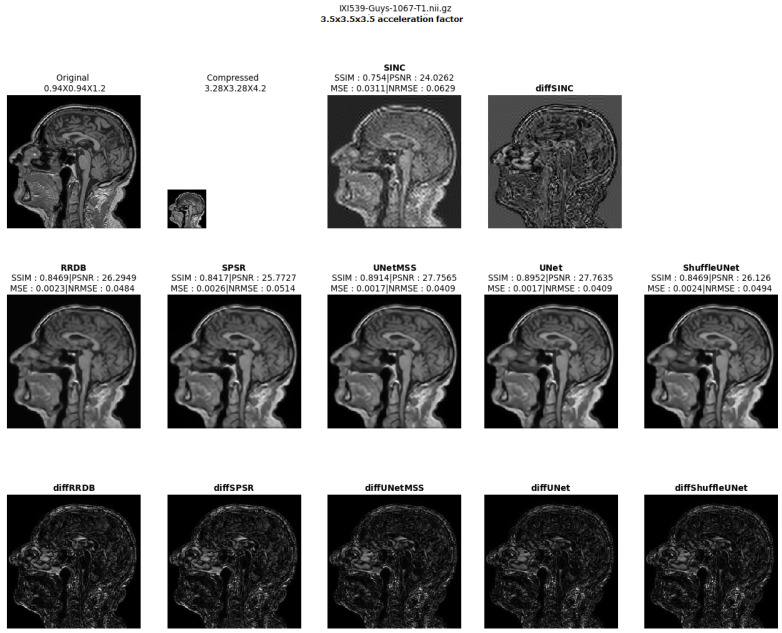
Model comparison for acceleration factor of $3.5^{3}$ (IXI-T1 dataset).

**Figure 11:**
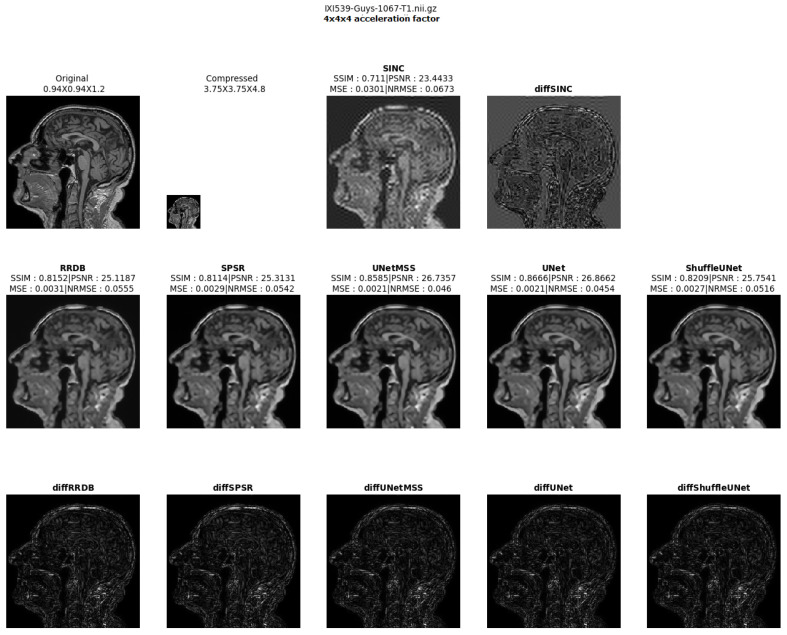
Model comparison for acceleration factor of $4^{3}$ (IXI-T1 dataset).

**Figure 12:**
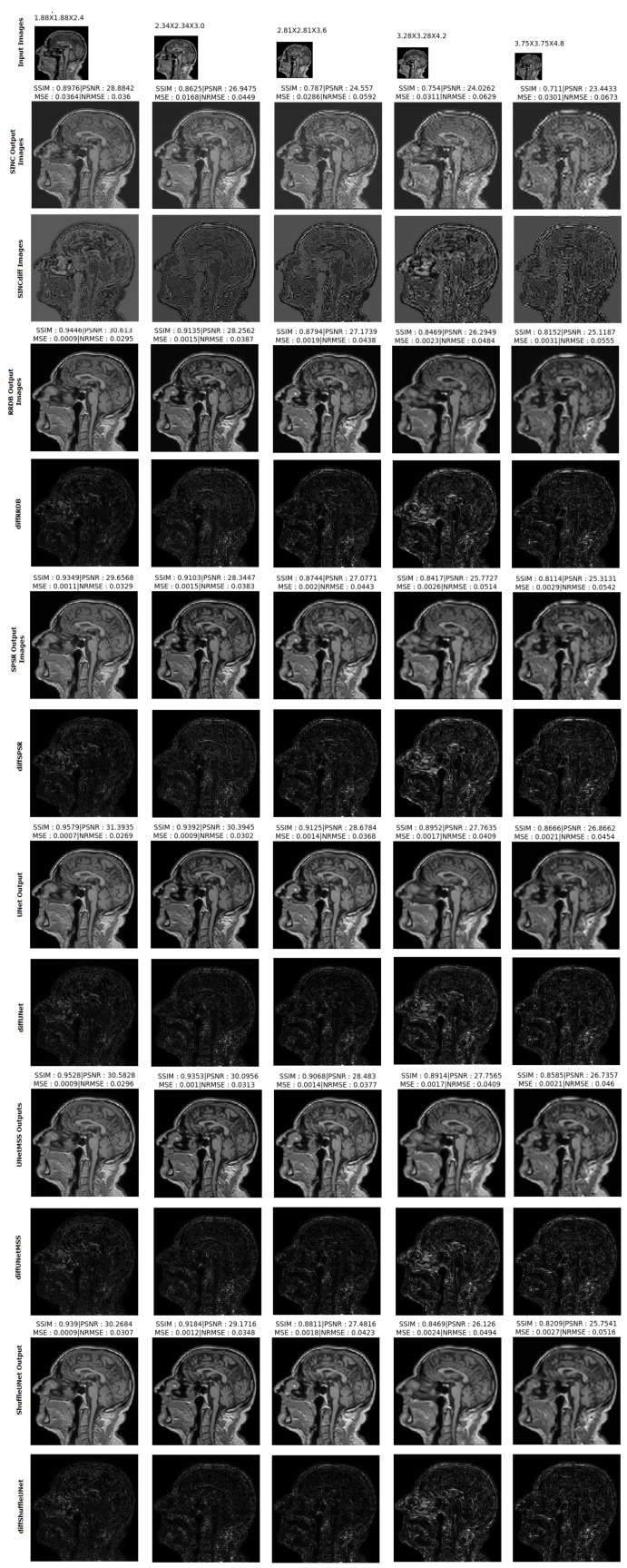
Comparison of the model outputs, along with the Sinc interpolated output, with the help of difference images and scores obtained on different metrics from IXI-T1 dataset.

The result comparison of models evaluated for individual acceleration factors can be found in Figure 7, Figure 8, Figure 9, Figure 10 and Figure 11. Figure 12 shows a complete comparison of different models and their performance for different acceleration factors. In addition, the values of the corresponding evaluation metrics can be found in Table 2 and Table 3. In each figure, the input image corresponding to the compressed image and the sinc-interpolated images are shown in the first row. The five model outputs and the difference images from respective ground truth are shown in the 2nd and 3rd rows.

**Figure 13:**
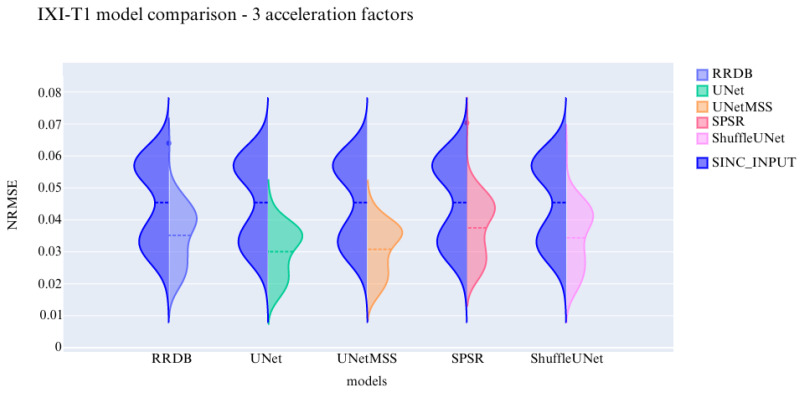
Comparison of the deep learning models using NRMSE for IXI-T1 dataset.

**Figure 14:**
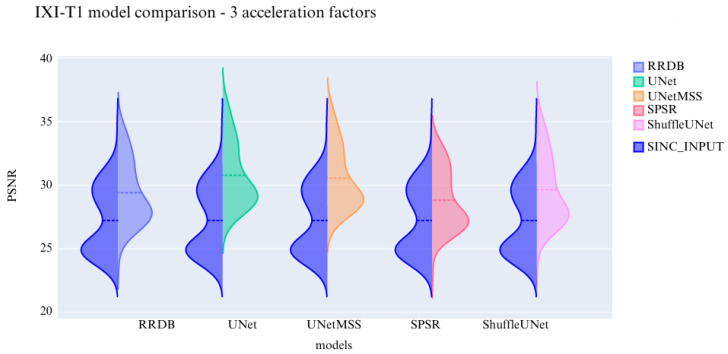
Comparison of the deep learning models using PSNR for IXI-T1 dataset.

**Figure 15:**
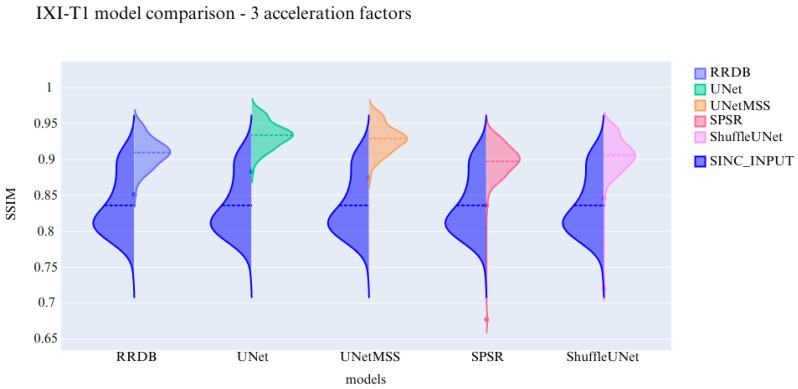
Comparison of the deep learning models using SSIM for IXI-T1 dataset.

The visualisation of the violin plot for model comparisons in different metrics for the acceleration factor ∼27 is shown in Figure 13, Figure 14 and Figure 15.

##### Models Comparison on Trainable Parameters and Inference Time

For choosing the best models for the main experiments for generalisability, inference time also played an important role. The authors considered models with a shorter inference time as this is more practical for real clinical applications. Table 4 presents a comparison of the models in terms of the number of trainable parameters and the time (in seconds) required to infer one volume. Unet/UnetMSS and RRDB were the simplest and low-inference-time models among all the models.

As the three best models were selected to go further for the main experimentation, independent T-tests were conducted to check the statistical significance between the pairs of these finalised models. It was found that:

1. RRDB with UnetMSS for the acceleration factor ∼27 resulted in a *p* value of 8.63 $\times \;10^{-14}$, which indicates that the difference is statistically significant.
2. UNet with UNetMSS for acceleration factor ∼27 resulted in a *p* value 0.0193, indicating that there is statistical difference between them.
3. *p* value increases with higher acceleration factor between UNet and UNetMSS. (All the table of *p* values can be found in the Appendix A.2).

From all of the analyses, it was concluded that for cross-acceleration factor trained models, UNet performs significantly better compared to all other models, this holds for all acceleration factors.

#### 3.1.2. Main Experiments and Model Comparison

From the results of the initial experiments, it was observed that the UNet, UNetMSS, ShuflleUNet and RRDB models performed better, as seen in Table 2 and Table 3. Among them, ShuffleUNet is resource-heavy and has a high inference time. Hence, only RRDB, UNetMSS and UNet were chosen for further experimentations.

These experiments are classified as:

1. Cross contrast experiments
2. Uncertainty mapping

##### Cross Contrast Experiments

Previous experiments were performed on scale generic models having all five discrete scale factors to evaluate the scale generalisability of the models. Now, the experiments are performed to test the models for their contrast generalising capabilities. For this part of the experiments, the complexity of the dataset was increased by including images from three different contrasts (T1, T2, and PD). All hyper parameters are kept the same for these 3 finalised models. The models are evaluated and then further compared using violin plots. For a common reference, all the violin plots represent the results from models on data of the 3 × 3 × 3 acceleration factor.

**Figure 16:**
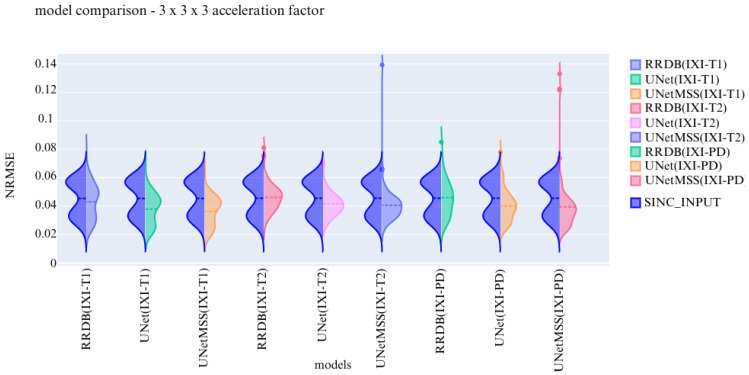
Model comparison on cross contrast for NRMSE.

**Figure 17:**
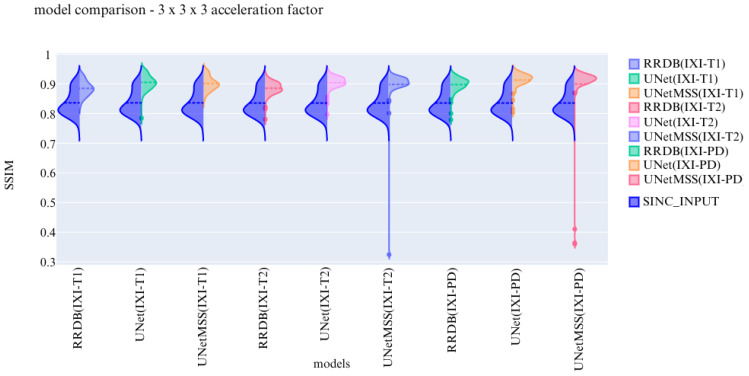
Model comparison on cross contrast for SSIM.

**Figure 18:**
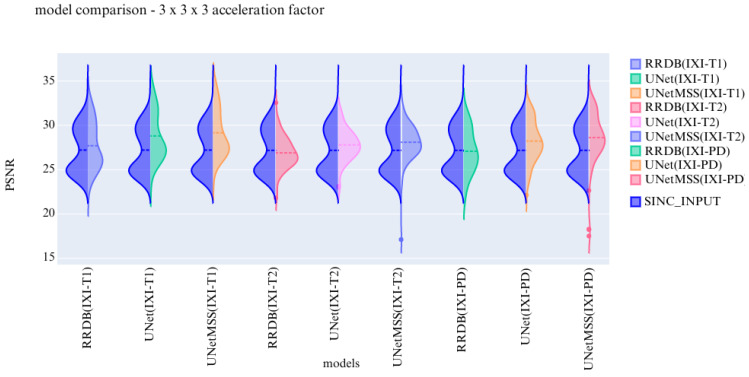
Model comparison on cross contrast for PSNR.

In the violin plot Figure 16, Figure 17 and Figure 18 RRDB, UNet, UNetMSS models trained for cross-contrast and evaluated for T1, T2, and PD are compared using SSIM, NRMSE and PSNR evaluation metrics.

The evaluation results for all the contrast and acceleration factors can be found in the Appendix A.2 Table A4.

##### Uncertainty Mapping

**Figure 19:**
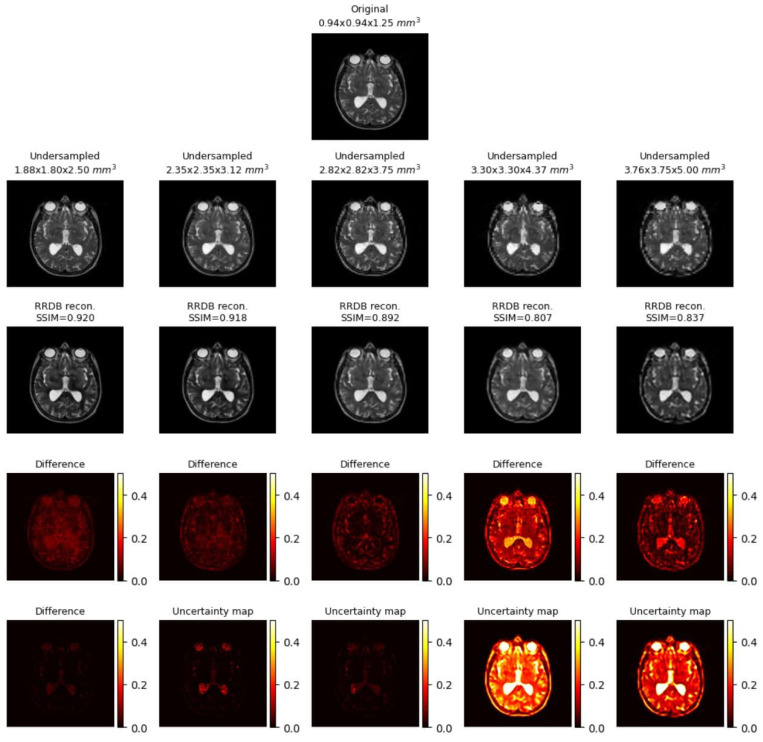
Sample uncertainty visualisations while RRDB model performed super-resolution across five discrete acceleration factors (2, 2.5, 3, 3.5, 4 along all three dimensions, theoretical acceleration factors of 8, 16, 27, 43, and 64, respectively), along with the difference images and the estimated uncertainty maps. The resultant SSIM values for the shown images are also reported. The difference images and the uncertainty maps were normalised for visualisation.

**Figure 20:**
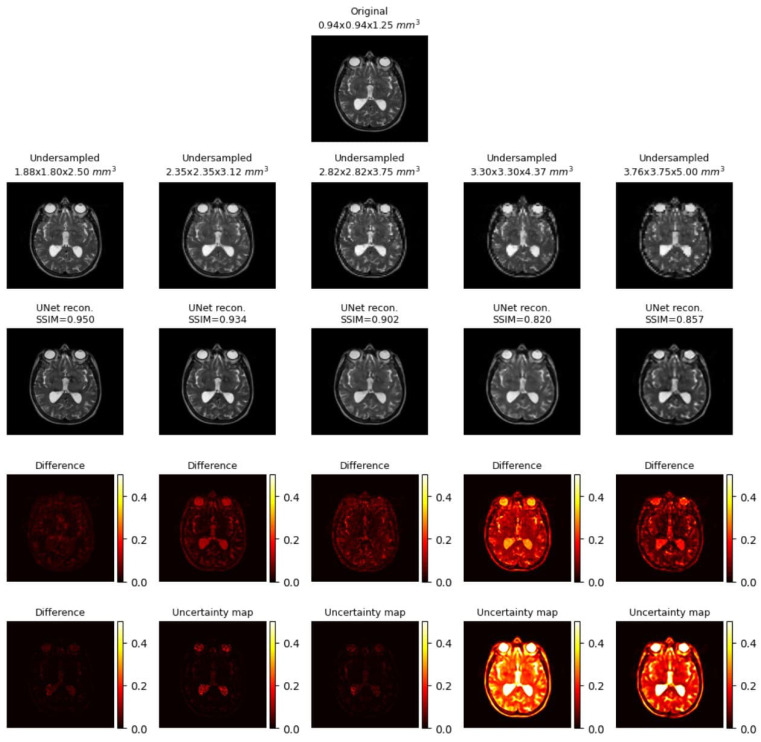
Sample uncertainty visualisations while UNet model performed super-resolution across five discrete acceleration factors (2, 2.5, 3, 3.5, 4 along all three dimensions, theoretical acceleration factors of 8, 16, 27, 43, and 64, respectively), along with the difference images and the estimated uncertainty maps. The resultant SSIM values for the shown images are also reported. The difference images and the uncertainty maps were normalised for visualisation.

**Figure 21:**
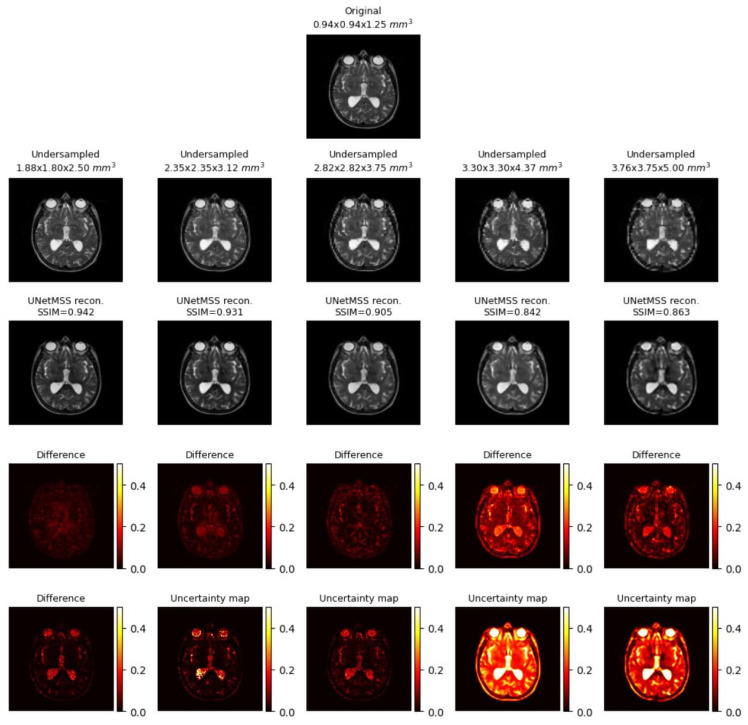
Sample uncertainty visualisations while UNetMSS model performed super-resolution across five discrete acceleration factors (2, 2.5, 3, 3.5, 4 along all three dimensions, theoretical acceleration factors of 8, 16, 27, 43, and 64, respectively), along with the difference images and the estimated uncertainty maps. The resultant SSIM values for the shown images are also reported. The difference images and the uncertainty maps were normalised for visualisation.

**Figure 22:**
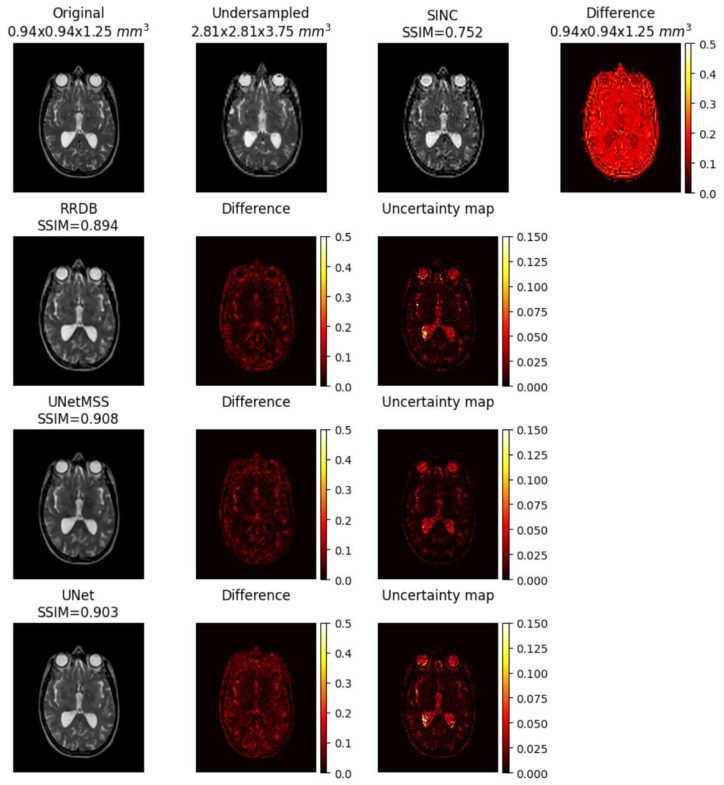
Uncertainty mapping for finalised models (IXI-T2 dataset).

**Figure 23:**
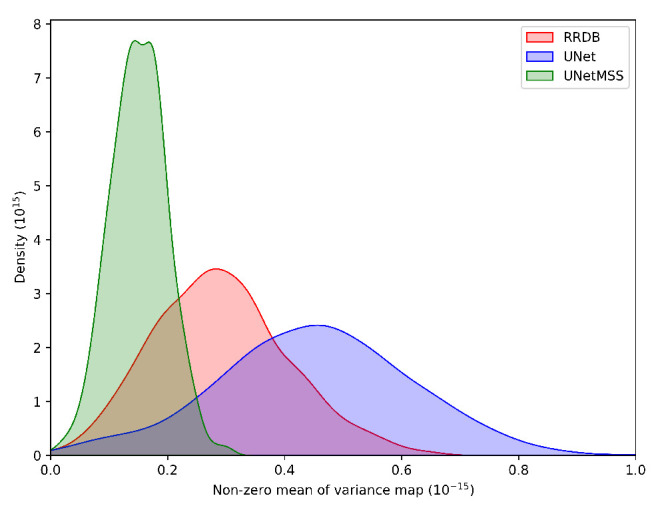
Histogram representing the distribution of non-zero mean of the variance map generated by the training free uncertainty mapping method.

As explained in Section 2.4, the uncertainty maps for RRDB, UNet and UNetMSS for individual contrast can be found in the Figure 19, Figure 20 and Figure 21, while Figure 22 shows a comprehensive comparison of these models in terms of reconstruction difference and uncertainty maps. Infer-noise and infer-dropout methods were evaluated on the dataset and estimated a pixel-wise variance mapping for these two methods. One method to evaluate the uncertainty estimation is to calculate the co-relation between the generated uncertainty maps and the L1 maps. 20 images were randomly sampled from the data set at the acceleration factor $3^{3}$, then used to generate the uncertainty maps, and finally a correlation analysis was performed with the L1 loss map of the images. However, it was observed that for all uncertainty maps generated from all images had a very low average pixel-wise variance of order $10^{-16}$. The KDE plot in Figure 23 shows the distribution of non-zero mean of the variance map generated by the three main models (RRDB, UNet and UNetMSS) on the 20 sample images.

### 3.2. Discussion

This paper demonstrated four different existing and one custom-built 3D deep learning super-resolution models for MRI using the IXI dataset for T1, T2, and PD contrasts. Also, by performing experiments with cross-contrast and cross-acceleration factor data, the authors evaluated the robustness of these models aiding with Uncertainty Mappings. We further showed the generalisation capabilities of these models. All models were trained with the same set of hyperparameters to allow for a fair comparison among them. All 3D deep learning models showed noticeable improvement qualitatively and quantitatively while reconstructing low-resolution data compared to traditional interpolation methods. Initial experiments are conducted with RRDB, SPSR, UNet, UNetMSS and ShuflleUNet architectures considering only single intensity IXI-T1 data. Evaluation and inference time were considered to choose the models for generalisation experiments. Although ShuflleUNet performance was considerable, it was ignored for further analyses due to its significant inference time of 44.10 s. Boosting the fact, ShuflleUNet trainable parameters are ten-times higher in reference with other models.

**Figure 24:**
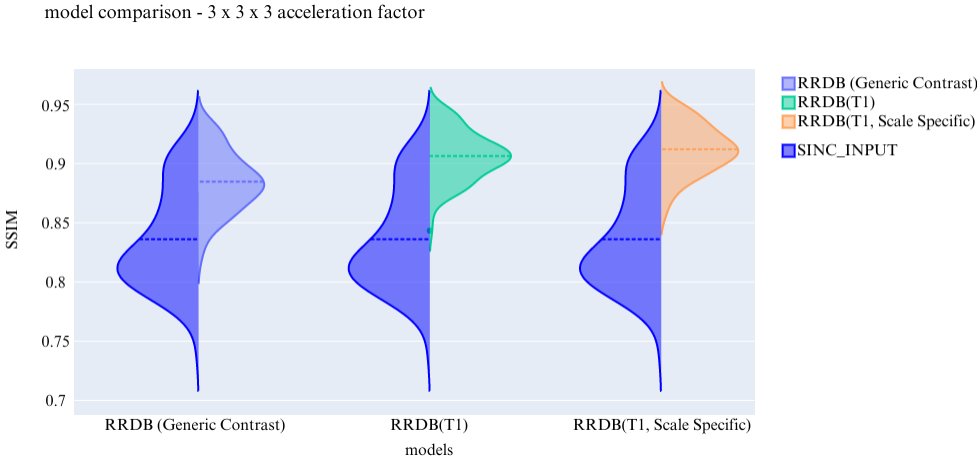
Violin plot showing SSIM metric for RRDB scale generalisability.

**Figure 25:**
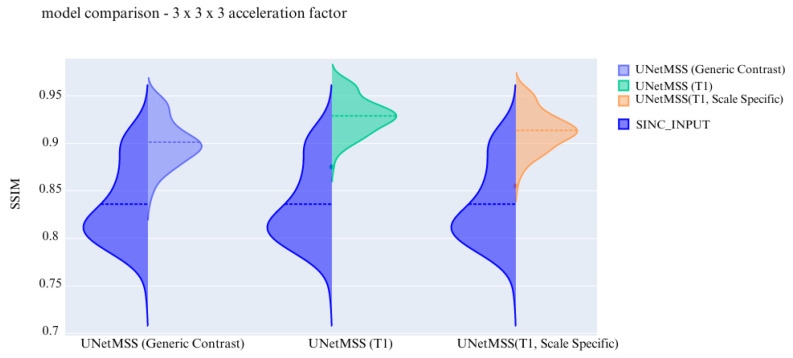
Violin plot showing SSIM metric for UNetMSS scale generalisability.

**Figure A6:**
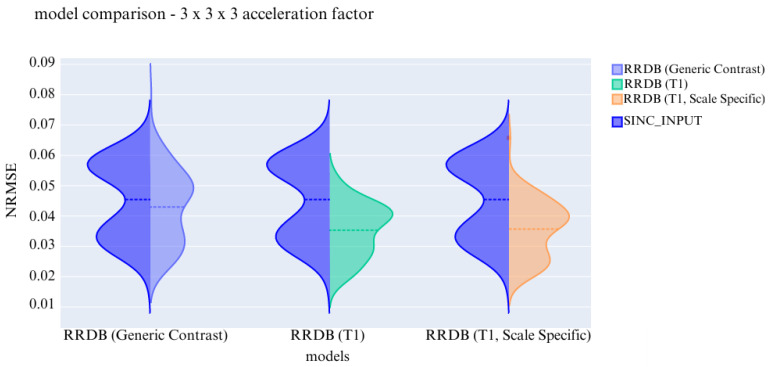
Violin plot showing NRMSE metric for RRDB scale generalisability.

**Figure A7:**
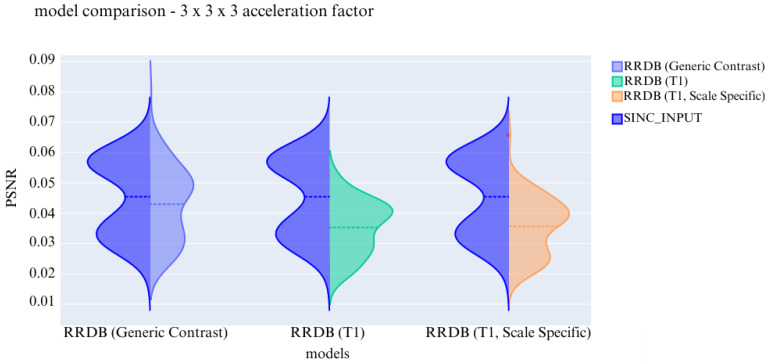
Violin plot showing PSNR metric for RRDB scale generalisability.

**Figure A8:**
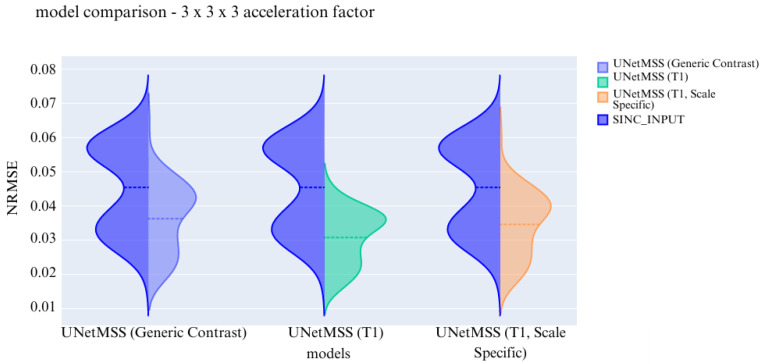
Violin plot showing NRMSE metric for UNetMSS scale generalisability.

**Figure A9:**
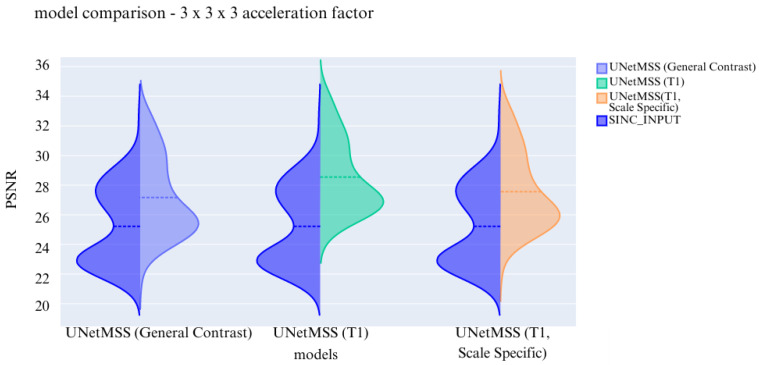
Violin plot showing PSNR metric for UNetMSS scale generalisability.

The models were compared according to the metrics on the generic models (T1, T2, and PD) to show the best performing model of all scenarios. From Figure 24, it was observed that the SSIM metric values decreased as and when the generalisability of the dataset increased. Model performance is comparatively good in acceleration factor-specific trained models. The trend follows the same for UNetMSS in Figure 25. All the comparative plots can be seen in Figure A6, Figure A7, Figure A8, Figure A9 and Figure 25.

From statistical analysis using independent T-test, the following conclusions could be drawn:

1. UNet and UNetMSS perform significantly better compared to RRDB in all the acceleration factors and contasts, except $2^{3}$ and T2.
2. UNet and UNetMSS are not significantly different in all acceleration factors.

Hence, UNet and UnetMSS were considered as the winning models. All independent T-test results (*p* values) can be found in the Appendix Table A4.

It is interesting to note that UNet and UNetMSS perform better than the newer models. The superior performance of these models in super-resolving undersampled MRIs, as compared to newer models such as SPSR, ShuffleUNet, and RRDB, can be attributed to several key factors. Firstly, the symmetric encoder–decoder structure of UNet, with its skip connections, enables robust localisation and effective integration of multi-scale contextual information, crucial for reconstructing fine details in medical images. UNetMSS further enhances this capability through multi-scale supervision, allowing the model to capture and synthesise information across varying resolutions. Moreover, the simpler architecture of these models offers a better bias-variance trade-off, reducing the risk of overfitting, particularly when working with limited or noisy datasets. Given that UNet was originally designed for tasks analogous to inverse problems, such as image segmentation, its architecture is inherently suited to reconstruct lost details from incomplete data. This is particularly important in the medical imaging domain, where these models have been extensively fine-tuned to handle the specific challenges of MRI data, such as varying contrast, noise, and anatomical variability. Consequently, UNet and UNetMSS not only generalise more effectively to unseen data but also exhibit faster convergence and greater robustness, making them particularly well-suited for practical applications in medical image super-resolution.

## 4. Conclusions and Future Work

### 4.1. Conclusions

This research aimed to compare various deep learning models for the purpose of super-resolution in MRI imaging. Initial experiments were conducted using DenseNet, employing the publicly available IXI-T1 dataset with a downsampling factor of two as the input image. Subsequently, the RRDB model was implemented as an extension of DenseNet. Following the acquisition of results from the RRDB model, attention was shifted to the UNet architecture. Simultaneously, experiments were conducted with various loss functions, including L1, SSIM, Perceptual L1, Perceptual SSIM, and MGL. Upon analysing the results, it was concluded that the models trained with the SSIM loss function outperformed those trained with other loss functions. The same methodology was then applied to the training of additional models, specifically UNetMSS, ShuffleUNet, and SPSR. A comparative analysis of these latter models with RRDB and UNet revealed that RRDB, UNet, and UNetMSS produced superior results compared to the other models. Based on the conclusions drawn from all experiments, RRDB, UNet, and UNetMSS were selected as the final models. In real-time clinical applications, multiple downsampling factors are employed depending on specific requirements. Therefore, the authors adopted a scale-generalised approach, ranging from 2 to 4, with increments of 0.5. The models were further trained using cross-contrast datasets, including T1, T2, and PD, to enhance generalisation and improve the robustness of the models. Finally, the models were evaluated and compared based on various evaluation metrics. To help users assess the robustness of the models and evaluate the reconstructions during run-time, an uncertainty mapping pipeline was also developed, and the models were evaluated for their uncertainty.

### 4.2. Future Work

- This research used discrete acceleration factors to downsample the images and then pre-interpolated them before training the models. It could have been avoided by pre-interpolation and upscale directly on the model training pipeline, so that the model can dynamically super-resolve any Acceleration factor.
- Diffusion models [29] are considered to be current state-of-the-art models, where a model is trained to add noise to a low-resolution image and then perform super-resolution by denoising these low-resolution images. This method recently proved to provide good results for images from very low resolution.
- Test block-wise uncertainty mapping by applying a local segmentation algorithm, since they gave good loss-variance correlation in the work done by [27].
- Deep learning models are prone to security concerns, such as adversarial attacks. Such security concerns were out of the scope of the current research, but should be considered as a future research direction [30,31].

## Figures and Tables

**Table 1:** Number of subjects in each individual data split.

Dataset	Training	Validation	Test
IXI-T1	406	70	105
IXI-T2	403	70	104
PD	403	70	104

**Table 2:** SSIM values of different methods (interpolation methods and deep learning models) on IXI-T1 dataset.

Method Type	Method Name	Acceleration Factor				
		2^3^	2.5^3^	3^3^	3.5^3^	4^3^
Interpolation Methods (non-DL)	Bicubic	0.90 ± 0.0223	0.87 ± 0.032	0.81 ± 0.0412	0.78 ± 0.0414	0.73 ± 0.0477
	NN	0.89 ± 0.0245	0.83 ± 0.0371	0.77 ± 0.0461	0.74 ± 0.0448	0.69 ± 0.0496
	Sinc	0.93 ± 0.0239	0.89 ± 0.0252	0.83 ± 0.0409	0.80 ± 0.0407	0.77 ± 0.0461
Deep Learning Models	RRDB	0.95 ± 0.0084	0.93 ± 0.0141	0.91 ± 0.0206	0.88 ± 0.0255	0.86 ± 0.0295
	SPSR	0.91 ± 0.0118	0.91 ± 0.0124	0.87 ± 0.0139	0.84 ± 0.0208	0.81 ± 0.0255
	UNet	**0.97 ± 0.0087**	**0.95 ± 0.0121**	**0.93 ± 0.0179**	**0.92 ± 0.0202**	**0.90 ± 0.0251**
	UNetMSS	0.96 ± 0.0111	0.94 ± 0.0138	0.92 ± 0.0206	0.90 ± 0.0216	0.88 ± 0.0269
	ShuffleUNet	0.95 ± 0.0112	0.94 ± 0.0140	0.90 ± 0.0479	0.88 ± 0.0246	0.88 ± 0.0412

**Table 3:** NRMSE values of different methods (interpolation methods and deep learning models) on IXI-T1 dataset.

Method Type	Method Name	Acceleration Factor				
		2^3^	2.5^3^	3^3^	3.5^3^	4^3^
Interpolation Methods (non-DL)	Bicubic	0.0378 ± 0.0095	0.0437 ± 0.0121	0.0544 ± 0.0149	0.0577 ± 0.0148	0.0676 ± 0.0153
	NN	0.0418 ± 0.0109	0.0522 ± 0.0139	0.0643 ± 0.017	0.0665 ± 0.0165	0.0763 ± 0.0174
	Sinc	0.0273 ± 0.0086	0.0350 ± 0.009	0.0454 ± 0.0125	0.0495 ± 0.0124	0.0523 ± 0.0142
Deep Learning Models	RRDB	0.026 ± 0.005	0.0302 ± 0.0004	0.0352 ± 0.0086	0.0394 ± 0.0093	0.0429 ± 0.011
	SPSR	0.039 ± 0.0041	0.038 ± 0.0073	0.045 ± 0.0095	0.051 ± 0.0124	0.054 ± 0.0136
	UNet	**0.021 ± 0.0050**	**0.025 ± 0.0063**	**0.03 ± 0.0082**	**0.035 ± 0.0084**	**0.036 ± 0.0089**
	UNetMSS	0.024 ± 0.0052	0.029 ± 0.0065	0.034 ± 0.0086	0.038 ± 0.0083	0.04 ± 0.0087
	ShuffleUNet	0.026 ± 0.0072	0.032 ± 0.0093	0.036 ± 0.0195	0.043 ± 0.0115	0.046 ± 0.0114

**Table 4:** Comparison of model trainable parameters and inference time.

Model	Trainable Parameters	Inference Time (in Seconds)
RRDB	246,865	8.40
SPSR	493,754	18.86
UNet/UNetMSS	5,418,563	8.79
ShuffleUNet	106,957,377	44.10

**Figure A1:**
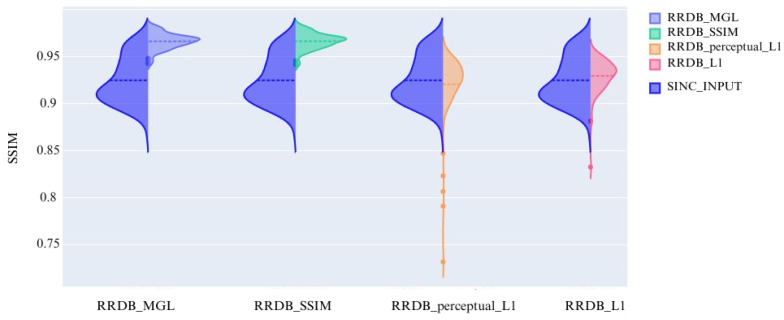
SSIM metric comparison for loss functions.

**Figure A2:**
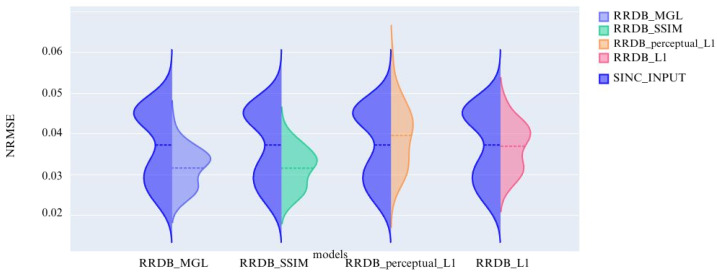
NRMSE metric comparison for loss functions.

**Figure A3:**
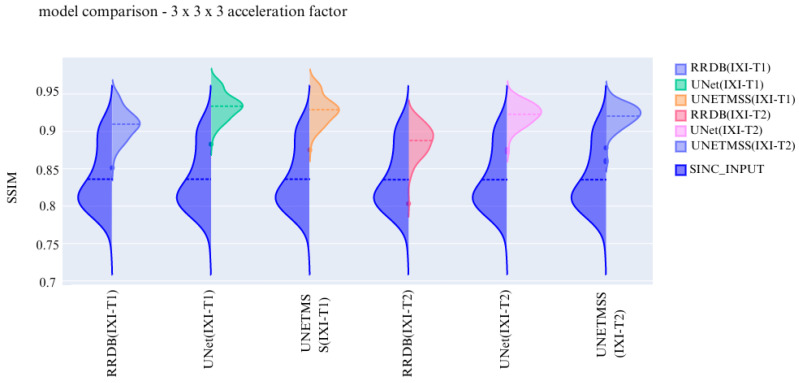
Model comparison on different contrast for SSIM.

**Figure A4:**
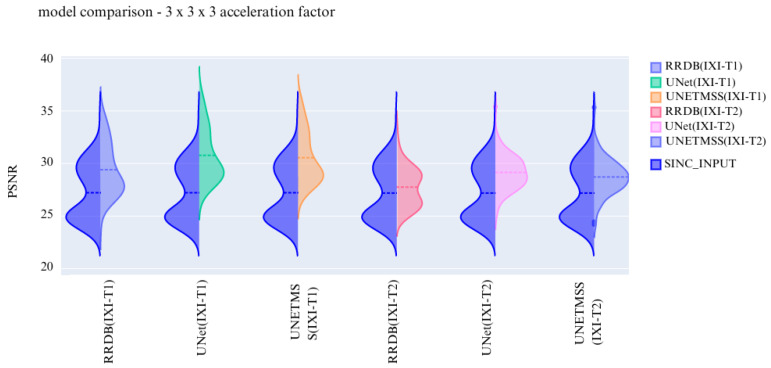
Model comparison on different contrast for PSNR.

**Figure A5:**
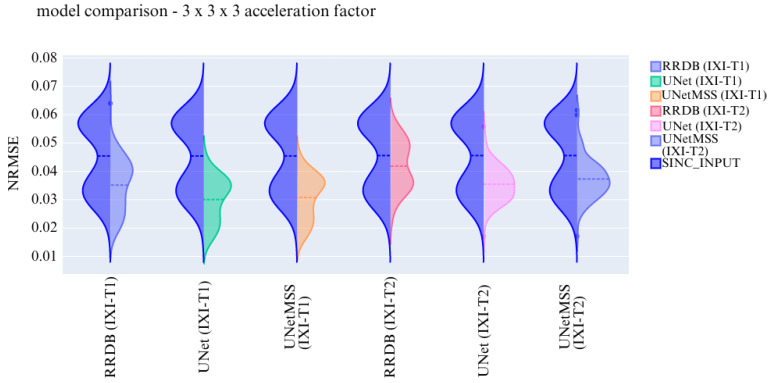
Model comparison on different contrast for NRMSE.

## Data Availability

This research used publicly available IXI dataset that can be downloaded from: https://brain-development.org/ixi-dataset/ (accessed on 17 August 2024). The code is available online at: https://github.com/venkatesh-thiru/SuperRes (accessed on 17 August 2024). Some of the weights are available as a collection on huggingface: https://huggingface.co/collections/venkatesh-thiru/beyond-nyquist-a-comparative-analysis-of-3d-deep-learning-m-66c26c1dcb6aab077492fec3 (accessed on 17 August 2024).

## Appendix A

### Appendix A.1. Results Comparison of Different Loss Functions

The authors trained the RRDB model with five different loss functions, namely SSIM, L1, perceptual L1 for an acceleration factor of eight with IXI-T1 dataset. The results for them can be found in Table A1. Further experiments were carried out with the SSIM loss function. The comparison of models in the SSIM, NRMSE, and PSNR evaluation metrics for different loss functions can be found Figure A1 and Figure A2. Independent T-tests were performed on the SSIM metrics on models trained with MGL and SSIM loss functions, obtained a *p*-value of 0.3676 which proves that the difference between them is insignificant. The authors chose to continue to experiment with the SSIM loss function, since the MGL also works with SSIM as a criterion to compare the images and the gradients of the images. Table A1 provides all the mean values for different loss functions for the respective evaluation metrics.

**Table A1:** Loss function comparison.

Loss Function	Evaluation Metric		
	SSIM	PSNR	NRMSE
MGL	0.9664 ± 0.0084	33.5291 ± 2.0137	0.0216 ± 0.0048
SSIM	**0.9666 ± 0.0085**	**33.5574 ± 2.0781**	**0.0216 ± 0.0223**
L1	0.9297 ± 0.0176	31.5964 ± 1.9482	0.0270 ± 0.0058
Perceptual L1	0.9250 ± 0.0240	31.8038 ± 3.1137	0.0273 ± 0.0088

### Appendix A.2. Main Experiments

In the below violin plot figures, Figure A3, Figure A4 and Figure A5 RRDB, UNet, UNetMSS models trained for cross contrast and evaluated for IXI-T1, IXI-T2 and IXI-PD are compared for SSIM, PSNR, NRMSE evaluation metrics.

**Table A2:** Independent T-test for Unet and UnetMSS on IXI-T1 dataset.

Acceleration Factor	*p*-Value
2^3^	0.0193
2.5^3^	0.0552
3^3^	0.0735
3.5^3^	0.1464
4^3^	0.195

**Table A3:** Evaluation metrics on scale-contrast generic models.

Contrast	AF (^3)	Model	NRMSE		SSIM	
			Mean	Std	Mean	Std
IXI-T1	2	RRDB	0.0298	0.0057	0.9422	0.0114
	2	SINC_INPUT	0.0273	0.0088	0.9250	0.0239
	2	UNet	0.0293	0.0080	0.9476	0.0113
	2	UNetMSS	0.0281	0.0053	0.9438	0.0142
	2d5	RRDB	0.0366	0.0086	0.9164	0.0151
	2d5	SINC_INPUT	0.0351	0.0091	0.8947	0.0253
	2d5	UNet	0.0328	0.0089	0.9308	0.0141
	2d5	UNetMSS	0.0326	0.0067	0.9228	0.0172
	3	RRDB	0.0430	0.0122	0.8847	0.0249
	3	SINC_INPUT	0.0454	0.0126	0.8361	0.0409
	3	UNet	0.0379	0.0107	0.9049	0.0256
	3	UNetMSS	0.0357	0.0089	0.9041	0.0212
	3d5	RRDB	0.0487	0.0125	0.8612	0.0268
	3d5	SINC_INPUT	0.0495	0.0125	0.8060	0.0407
	3d5	UNet	0.0420	0.0105	0.8902	0.0235
	3d5	UNetMSS	0.0387	0.0096	0.8874	0.0251
	4	RRDB	0.0538	0.0127	0.8307	0.0336
	4	SINC_INPUT	0.0524	0.0143	0.7676	0.0462
	4	UNet	0.0458	0.0101	0.8638	0.0307
	4	UNetMSS	0.0421	0.0104	0.8643	0.0298
IXI-T2	2	RRDB	0.0292	0.0098	0.9441	0.0230
	2	SINC_INPUT	0.0263	0.0064	0.9339	0.0144
	2	UNet	0.0309	0.0127	0.9439	0.0409
	2	UNetMSS	0.0260	0.0072	0.9496	0.0179
	2d5	RRDB	0.0360	0.0074	0.9223	0.0149
	2d5	SINC_INPUT	0.0342	0.0073	0.9029	0.0168
	2d5	UNet	0.0339	0.0080	0.9334	0.0165
	2d5	UNetMSS	0.0321	0.0066	0.9298	0.0166
	3	RRDB	0.0461	0.0095	0.8852	0.0221
	3	SINC_INPUT	0.0490	0.0110	0.8373	0.0238
	3	UNet	0.0415	0.0085	0.9038	0.0224
	3	UNetMSS	0.0381	0.0080	0.9061	0.0187
	3d5	RRDB	0.0506	0.0106	0.8588	0.0231
	3d5	SINC_INPUT	0.0541	0.0129	0.8073	0.0278
	3d5	UNet	0.0451	0.0102	0.8880	0.0218
	3d5	UNetMSS	0.0438	0.0093	0.8811	0.0215
	4	RRDB	0.0572	0.0166	0.8289	0.0654
	4	SINC_INPUT	0.0555	0.0127	0.7873	0.0279
	4	UNet	0.0506	0.0154	0.8569	0.0648
	4	UNetMSS	0.0477	0.0097	0.8627	0.0289
PD	2	RRDB	0.0278	0.0101	0.9546	0.0119
	2	SINC_INPUT	0.0229	0.0062	0.9423	0.0137
	2	UNet	0.0308	0.0125	0.9561	0.0132
	2	UNetMSS	0.0242	0.0074	0.9579	0.0111
	2d5	RRDB	0.0364	0.0099	0.9322	0.0142
	2d5	SINC_INPUT	0.0313	0.0075	0.9111	0.0176
	2d5	UNet	0.0345	0.0092	0.9412	0.0127
	2d5	UNetMSS	0.0276	0.0065	0.9419	0.0130
	3	RRDB	0.0460	0.0128	0.8976	0.0247
	3	SINC_INPUT	0.0474	0.0126	0.8502	0.0242
	3	UNet	0.0401	0.0106	0.9128	0.0238
	3	UNetMSS	0.0347	0.0089	0.9179	0.0177
	3d5	RRDB	0.0579	0.0162	0.8741	0.0202
	3d5	SINC_INPUT	0.0528	0.0153	0.8196	0.0282
	3d5	UNet	0.0491	0.0143	0.8972	0.0189
	3d5	UNetMSS	0.0427	0.0110	0.8983	0.0171
	4	RRDB	0.0598	0.0188	0.8447	0.0625
	4	SINC_INPUT	0.0513	0.0140	0.8055	0.0299
	4	UNet	0.0491	0.0145	0.8705	0.0542
	4	UNetMSS	0.0433	0.0101	0.8844	0.0206

**Table A4:** Independent T-Test on scale-contrast generic models on SSIM metric.

Contrast	AF	Comparison	*p* Value
IXI-T1	8	RRDB vs. UNetMSS	0.1172
		RRDB vs. UNet	0
		RRDB vs. SINC_INPUT	0
		UNetMSS vs. UNet	0.0002
		UNetMSS vs. SINC_INPUT	0
	27	RRDB vs. UNetMSS	0
		RRDB vs. UNet	0
		RRDB vs. SINC_INPUT	0
		UNetMSS vs. UNet	0.6798
		UNetMSS vs. SINC_INPUT	0
	64	RRDB vs. UNetMSS	0
		RRDB vs. UNet	0
		RRDB vs. SINC_INPUT	0
		UNetMSS vs. UNet	0.8103
		UNetMSS vs. SINC_INPUT	0
IXI-T2	2	RRDB vs. UNetMSS	0.0009
		RRDB vs. UNet	0.9546
		RRDB vs. SINC_INPUT	0
		UNetMSS vs. UNet	0.0253
		UNetMSS vs. SINC_INPUT	0
	3	RRDB vs. UNetMSS	0
		RRDB vs. UNet	0
		RRDB vs. SINC_INPUT	0
		UNetMSS vs. UNet	0.1721
		UNetMSS vs. SINC_INPUT	0
	4	RRDB vs. UNetMSS	0
		RRDB vs. UNet	0
		RRDB vs. SINC_INPUT	0
		UNetMSS vs. UNet	0.1562
		UNetMSS vs. SINC_INPUT	0
PD	2	RRDB vs. UNetMSS	0.0005
		RRDB vs. UNet	0.1346
		RRDB vs. SINC_INPUT	0
		UNetMSS vs. UNet	0.0747
		UNetMSS vs. SINC_INPUT	0
	3	RRDB vs. UNetMSS	0
		RRDB vs. UNet	0
		RRDB vs. SINC_INPUT	0
		UNetMSS vs. UNet	0.0027
		UNetMSS vs. SINC_INPUT	0
	4	RRDB vs. UNetMSS	0
		RRDB vs. UNet	0
		RRDB vs. SINC_INPUT	0
		UNetMSS vs. UNet	0
		UNetMSS vs. SINC_INPUT	0
